# Single-Target Implicit Association Tests (ST-IAT) Predict Voting Behavior of Decided and Undecided Voters in Swiss Referendums

**DOI:** 10.1371/journal.pone.0163872

**Published:** 2016-10-12

**Authors:** Livio Raccuia

**Affiliations:** Department of Political Science, University of Zurich, Zurich, Switzerland; Universitat de Valencia, SPAIN

## Abstract

Undecided voters represent a major challenge to political pollsters. Recently, political psychologists have proposed the use of implicit association tests (IAT) to measure implicit attitudes toward political parties and candidates and predict voting behavior of undecided voters. A number of studies have shown that both implicit and explicit (i.e., self-reported) attitudes contribute to the prediction of voting behavior. More importantly, recent research suggests that implicit attitudes may be more useful for predicting the vote of undecided voters in the case of specific political issues rather than elections. Due to its direct-democratic political system, Switzerland represents an ideal place to investigate the predictive validity of IATs in the context of political votes. In this article, I present evidence from three studies in which both explicit and implicit measures were used ahead of the vote on four different referendums. Explicit measures predicted voting better than implicit attitudes for decided voters while implicit and explicit attitudes were equally good predictors among undecided voters. In addition, implicit attitudes predicted voting behavior descriptively, but not significantly better for undecided voters while, also from a descriptive point of view, explicit attitudes predicted voting better for decided respondents. In sum, results suggest that, as argued in previous research, the predictive value of implicit attitudes may be higher in the context of issue-related votes but still not as high as initially hoped-for.

## Introduction

*Perhaps the most compelling way for implicit social cognition to establish its relevance to the study of politics is to enhance researchers’ ability to predict political behavior. (p. 558)* [1]

Explaining the political behavior of people lies at the heart of political research. In fact, opinion and exit polls have become an indispensable part of contemporary democracies. However, when asked to indicate their voting intention, people may either reveal which party or candidate they plan to vote for, or they may report themselves as being undecided. Irrespective of whether they are in fact undecided or simply unwilling to report their voting intention, undecided voters represent a major source of uncertainty when it comes to predicting the outcome of an election or vote. For this reason, political scientists have been looking for ways to improve polling accuracy by allocating undecided voters to the respective candidates, parties, or political camps. While many of these attempts have been based on using voter registration data [2], more recent attempts have focused on using implicit attitudes in order to improve the measurement and prediction of political behavior. Unlike explicit (i.e., self-reported) attitudes, implicit attitudes are more likely to operate below conscious awareness. In fact, previous research (e.g., [3]) has demonstrated that although people may be aware of their implicit attitudes, they may not be aware of (and thus not necessarily endorse) the ways in which their implicit preferences may impact their behavior. Hence, in the political realm, individuals’ implicit attitudes toward parties or candidates may affect their eventual voting behavior beyond their explicit attitudes. To date, the most widely used method to measure implicit attitudes is the implicit association test (IAT) [4]. While IATs have been used in many different domains, they were found to perform particularly well for the prediction of political behavior [5]. In fact, IATs were shown to provide predictive validity over and above explicit measures in a number of studies conducted ahead of political elections [6–8]. Therefore, evidence strongly suggests that political pollsters should take into account both explicit and implicit measures when concerned with the prediction of voting behavior.

In this article, I build on these and more recent findings [9, 10] suggesting that IATs may be more useful for the prediction of voting behavior in the case of specific political issues (rather than political elections) as a result of less elaborated attitudes toward these issues. If this is true, then IATs should be useful tools for the prediction of political outcomes in Switzerland, where people frequently decide on issue-related referendums. In contrast to political elections where voting behavior is typically determined by long-lasting party affiliations, voting on referendums represents a much more complex situation. In Swiss federal votes, coalitions often span across traditional party lines and voters frequently deal with highly complex issues. As a result, attitudes toward these issues most likely tend to be less elaborated than attitudes toward political parties and candidates. Somewhat surprisingly, however, no efforts have yet been made to investigate the predictive validity of IATs for vote outcomes in Swiss referendums. For this reason, I have first assessed the predictive validity of different types (traditional computer vs. computer-administered paper-format) of single-target implicit association tests (ST-IAT) in two studies that were conducted ahead of votes on such diverse issues as the purchase of fighter jets, minimum wage implementation, and public health insurance. In a second step, I have investigated whether implicit attitudes can add to the explanation of voting behavior of decided and undecided voters in the case of a very controversial referendum on immigration. All three studies were conducted online. In what follows, I will outline the most recent findings on the use of implicit attitudes for the prediction of political behavior and then present evidence from all three studies.

### Using Implicit Attitude Measures for the Prediction of Political Behavior

At first, implicit measures were seen as fit instruments to predict spontaneous, uncontrolled behavior, while rather unfit for predicting behavior in situations where people engage in deliberation and thus exert control over their behavior (e.g., [11]). As such, the value of IATs was deemed rather low for the prediction of political behavior. However, the focus soon shifted to mixture or additive models in which both explicit and implicit measures were believed to uniquely contribute to the explanation of (political) behavior [12]. In the political domain, Karpinski and colleagues [13] were first to use IAT scores to predict voting intention in the 2000 U.S. presidential election. They found IAT scores to be a significant predictor of voting intention. However, implicit attitudes no longer predicted voting intention once they controlled for explicit attitudes such as self-reported liking of the two candidates. Concerning the prediction of actual voting behavior, Friese et al. [6] used IATs for the first time in the lead-up to the 2002 parliamentary elections in Germany and found incremental predictive validity of Single-Target IATs (ST-IAT) for the five major political parties. Although self-reported party preferences were stronger predictors of voting intention and actual voting behavior, the implicit measures added significantly to the explanation of voting behavior. Hence, their findings were the first evidence in favor of additive prediction models in the domain of politics. In yet another study conducted in the lead-up to national elections, Arcuri and colleagues [7] also found predictive validity of IATs. More importantly, they found IATs to be significant predictors of voting behavior among both decided and undecided voters. Their findings were highly intriguing, as they increased optimism in the use of implicit measures for voters who do not − for whatever reason − report their voting intention. In a parallel study, Galdi et al. [9] first tested the utility of IATs in predicting future opinion and choice in the case of a local political issue in Italy. They found that for participants who were initially undecided, future (i.e., one week later) opinion about the enlargement of a U.S. military base was significantly predicted by a measure obtained in a Single-Category IAT (SC-IAT) [14]. Explicit attitudes, however, did not predict future opinions. Interestingly, for decided subjects, the pattern was exactly reversed. Against the background of their results, Galdi and colleagues [9] discussed convincing explanations for why implicit attitudes may be particularly useful for predicting the behavior of undecided individuals: while in the process of decision-making, the implicit attitude leads individuals to selectively expose themselves to information that corresponds with their implicit attitudes. As a result of the biasing influence of implicit attitudes, undecided voters may eventually develop a conscious (i.e., explicit) preference for one political party or candidate and this may then determine their ultimate vote decision. In addition, implicit attitudes may also predict voting behavior independent of their role in forming explicit attitudes. For example, implicit attitudes may lead to biased interpretations of information such as voting options even at the time of the vote. Interestingly, the authors successfully demonstrated their biased-processing account in a follow-up study [15].

In two recent studies, Roccato and Zogmaister [8] and Friese et al. [10] used IATs in the context of either national or presidential elections in Italy, Germany, and the United States. While both studies report evidence in favor of the additive prediction model, Friese and colleagues [10] found that explicit attitudes were better predictors of actual vote choice for both decided and undecided voters, while implicit attitudes were better predictors for the voting behavior of decided rather than undecided voters. As these findings are in contrast to Galdi et al.’s [9] results, the authors seek an explanation by pointing to the moderating role of cognitive elaboration. They argue that high cognitive elaboration will be reflected in high consistency (i.e., high overlap) between the implicit and explicit measure (see also [8, 13, 16] for this argument). As a consequence, it will be difficult for the implicit measure to predict incrementally over the explicit measure. Different study results may thus be explained by different levels of cognitive elaboration. For example, elaboration of attitudes may be lower in the case of specific political issues as in the Galdi et al. [9] study but rather high in the case of political party or candidate preferences. Friese et al. [10] indeed show that correlations between their implicit and explicit measures were much stronger than in Galdi and colleagues’ study [9]. Finally, Lundberg and Payne [17] used the Affect Misattribution Procedure (AMP) to capture implicit attitudes and a more precise measure of decidedness than the typically used dichotomous operationalization. Concretely, they used a continuous measure for confidence in one’s voting intention (1 = “not sure at all” to 5 = “extremely sure”), finding that at high levels of confidence, explicit attitudes are much stronger predictors than implicit attitudes, while at low levels of confidence, implicit and explicit attitudes are equally predictive of voting behavior. Moreover, Lundberg and Payne [17] provide a strong argument for why implicit attitudes may help to foresee the behavior of undecided voters: they show that confidence in one’s vote is a function of the strength of one’s explicit attitude toward the party or candidate. Hence, when individuals report themselves as either decided or undecided, they draw upon their explicit attitudes, neglecting their implicit attitudes. This, in turn, explains why explicit measures were found to be better predictors for the voting behavior of decided voters [9, 10, 17], while implicit measures were found to be good predictors for the voting behavior of both decided and undecided voters [7, 17].

Taken together, there is converging evidence [6, 8, 10, 17] in favor of additive prediction models that take into account both explicit and implicit measures. In other words, both explicit and implicit measures should be considered when predicting the outcome of political elections or votes. However, there is also convincing evidence on when implicit attitudes may be particularly useful and when they are not. In several studies [8, 10, 13, 17] conducted before political elections, the incremental predictive validity of IATs was relatively small as a result of rather high correlations between explicit and implicit measures. As discussed in Friese et al. [10], high consistency between the two types of measures may be the result of high cognitive elaboration. Because party or candidate preferences are well-elaborated attitudes, (ST-)IATs may be less attractive for predictions in the case of political elections. Yet, they may still be useful to predict voting behavior, particularly among undecided voters, in the case of specific political issues [9]. Due to its direct democratic political system, Switzerland provides a perfect setting for testing the predictive validity of (ST-)IATs in the context of national referendums. To my knowledge, however, no efforts have been made as yet in this direction. For all these reasons, I first examined two different types of ST-IATs and then selected one of them to assess its predictive validity for both decided and undecided voters in the case of a crucial referendum in Switzerland.

## Study 1

The first study was conducted between April 28, 2014 and May 7, 2014 and included two ST-IATs for assessing respondents’ implicit attitudes toward two referendums: first, a popular initiative for implementing a national minimum wage, and second, a referendum about the purchase of 22 Gripen fighter jets worth 3.1 billion Swiss francs. The vote was held on May 18, 2014. Both referendums were rejected (minimum wage initiative: 76.3 percent; Gripen referendum: 53.4 percent). The main goal of Study 1 was to get a first hint on whether ST-IATs can be used for the prediction of voting behavior in Swiss referendums. To this end, a small student sample was deemed sufficient and no power analysis was conducted to determine sample size. Instead, the survey was kept active until no additional responses were registered. Furthermore, because a pretest had shown that correspondence between voting intention and voting behavior is high (*r* = .81), a one-wave design was chosen. That is, subjects were only surveyed before the vote and asked to report their voting intention or vote choice if they had already voted.

### Methods

#### Ethics statement

The study was conducted online. All subjects were older than 18 years of age. In the invitation, subjects received brief information about the topic (Swiss Federal Votes) and the duration of the study as well as the researcher involved and his affiliation. On the first page, subjects were informed about the measures involved (questionnaire and two implicit association tests). At the end of the study, subjects received contact information. All data was analyzed anonymously and no identifying data (e.g., names) were collected. At the author’s faculty (Faculty of Arts, University of Zurich) the IRB asks researchers to self-assess the ethical soundness of their research using a checklist and only seek approval from the IRB if one of the questions is answered affirmatively. This was not the case for the present study.

#### Participants

Participants were 268 (47.4% males, 52.6% females) students, doctoral students, and employees of the University of Zurich. They received an email invite and were told that the study was about the upcoming national votes. Furthermore, they were asked to participate only if they are entitled to vote. Subjects’ mean age was 30.90 years (SD = 10.03) and they received no compensation in exchange for their participation.

#### Procedure

The entire study was written and administered using the Inquisit software and hosted at http://www.millisecond.com. After completing a short questionnaire on socio-demographic questions and questions pertaining to political interest and partisanship, participants completed the two ST-IATs in randomized order.

#### Measures

Implicit attitudes toward the two referendums were measured using a similar ST-IAT as the one used in Friese et al. [6] (see also [18, 19]). However, unlike their ST-IAT, I used an additional block of 15 trials ahead of each critical block, leading to a total number of 50 trials for each combination (see Table 1). This procedure was chosen because of the results from a pretest in which the predictive validity of the ST-IAT was unsatisfactory due to an order effect that occurred as a result of the low number of trials. As in Friese et al. [6], however, block and stimulus order were kept constant across respondents with each respondent starting with the positive vs. negative AND referendum response set. As can be seen in Table 1, subjects started with a practice block of 20 trials in which they had to discriminate between positive and negative words. Next, they completed two blocks of 15 and 35 trials in which positive attributes were assigned to the left-key (i.e., “E”) and negative attributes and words representing the respective referendum were assigned to the right-key (i.e., “I”). Finally, in the fourth and fifth block, the combination was reversed such that positive words and political stimuli were assigned to the left-key, while the right-key was left for negative words. Both evaluative categories were represented by five words (e.g., joy, love, stink, poison) while the two referendums were represented by three pictures (e.g., campaign poster) and two text stimuli (e.g., Swiss army) (see S1 Appendix). For calculating the ST-IAT scores, I used the improved scoring algorithm D2 proposed by Greenwald et al. [20]. Thus, all trials with response latencies less than 400 ms or greater than 10 seconds were discarded from the analyses, and a built-in error penalty was used in the case of incorrect responses. The resulting score can take on values between −2 and +2 and has several advantages. First, it provides a measure for both the direction and strength of one’s implicit attitude. Second, it applies a penalty for respondent errors and thus for very low latencies produced when sorting errors are committed. Finally, by dividing the difference in block means by its corresponding pooled standard deviation, the algorithm takes the respondent’s variability of response latencies into account [20]. The order of the two ST-IATs was randomized across participants.

**Table 1:** Sequence of Trial Blocks in ST-IATs in Study 1.

ST-IAT	Block	Items assigned to left-key response	Items assigned to right-key response	No. of Trials
1	1	Positive words	Negative words	20
	2	Positive words	Negative words & Minimum wage initiative	15
	3	Positive words	Negative words & Minimum wage initiative	35
	4	Positive words & Minimum wage initiative	Negative words	15
	5	Positive words & Minimum wage initiative	Negative words	35
2	1	Positive words	Negative words	20
	2	Positive words	Negative words & Gripen referendum	15
	3	Positive words	Negative words & Gripen referendum	35
	4	Positive words & Gripen referendum	Negative words	15
	5	Positive words & Gripen referendum	Negative words	35

Notes: The ST-IAT score is based on data from blocks 2,3,4 and 5. Block order was fixed across participants. For stimuli presentation, a fixed random order was used.

In this very preliminary study, the explicit measure was an 11-point scale for political orientation (0 = *extreme left* to 10 = *extreme right*) with higher values indicating a more rightist placement. Although political orientation did not represent a self-reported evaluation of the respective referendums (and thus measured a somewhat different construct), it was a good starting point for comparing the predictive validity of implicit and explicit measures. In fact, left-right (or liberal-conservative) self placement is one of the most prominent political measures routinely used in political surveys. In addition, it has been repeatedly found to be a strong predictor of political voting behavior, accounting for up to 85 percent of its variance [21]. It indeed turned out that both voting for the minimum wage initiative (*r* = −.66, *p* < .001) and the Gripen referendum (*r* = .48, *p* < .001) were closely related to participants’ political orientation.

Voting intention was measured using the following question: “What do you plan to vote at (i) the popular initiative ‘for a national minimum wage’ (ii) the ‘Gripen referendum’?” Response options were ‘Yes’, ‘No’, ‘Don’t know’. Undecided participants (*N*_*Min*.*wage*_ = 26, *N*_*Gripen*_ = 17) were excluded from the analyses. This left 154 decided voters for the minimum wage initiative and 163 decided voters for the Gripen referendum. Intentions to vote against the referendum were coded as 0, and intentions to vote in favor of it were coded as 1.

Voting behavior was measured with the following question: ‘How did you vote at (i) the popular initiative ‘for a national minimum wage’ (ii) the ‘Gripen referendum’?” Response options were ‘Yes’, ‘No’, ‘Abstained’. For both referendums, 86 participants reported their vote choice (there were no abstentions). Votes against the referendum were coded as 0, and votes in favor of it were coded as 1.

### Results and Discussion

#### Preliminary analyses

For each ST-IAT, I calculated the Spearman-Brown corrected split-half reliability as the correlation between the ST-IAT score of blocks 2 and 4 with the ST-IAT score of blocks 3 and 5. This led to similar reliability estimates for the minimum wage initiative (*r* = .57) and the Gripen referendum (*r* = .52). In addition, I examined the correspondence between the implicit and explicit measure for both referendums. In previous research (e.g., [10, 13]), high implicit-explicit correspondence has been explained with well-elaborated attitudes. As a consequence, strong correlations between implicit and explicit attitudes were associated with high predictive validity of implicit attitudes overall but only little predictive value over and above explicit measures. Interestingly, I found only moderate correlations between the implicit and explicit measure for both the minimum wage initiative (*r* = −.40) and the Gripen referendum (*r* = .36). In addition, correlations did not significantly differ between participants who had already voted on the minimum wage initiative (*r* = −.48, *p* < .001) and those who had not yet voted but at least developed a voting intention (*r* = −.37, *p* < .001; *z* = −0.93, *p* of the difference between correlations = .35). In the case of the Gripen referendum, the correlations between the implicit and explicit measure were almost identical across the two groups of voters (Already voted: *r* = .33, *p* < .001, Decided: *r* = .38, *p* < .001; *z* = −0.39, *p* of the difference between correlations = .70). Taken together, these findings are consistent with the argument made in previous research (e.g., [10, 22]) that attitudes toward political issues are less elaborated than attitudes toward political parties or candidates.

#### Voting intention and voting behavior

Due to the one-wave design of the study, there is no data on undecided voters’ eventual voting decision. For this reason, I could not run a separate analysis on undecided voters. Therefore, in this very preliminary study, I cannot speak to the question of whether implicit attitudes are better predictors for undecided than decided voters. However, I can still assess the predictive value of implicit attitudes for the prediction of decided respondents’ voting intention and behavior in Swiss federal votes in general and beyond explicit measures.

Table 2 shows that for both referendums, implicit attitudes (models 1a) predicted voting intention. In fact, implicit attitudes allowed for the correct classification (% CCC) of 85.1 percent of (decided) voters in the case of the Gripen referendum, but they only predicted 62.8 percent of cases correctly in the case of the minimum wage initiative. The explicit measure (models 1b) outperformed the implicit measure on both occassions, correctly classifying 81.8 percent of cases (minimum wage initiative) and 85.3 percent of cases (Gripen referendum). Thus, it seems that consistent with previous research (e.g., [10]), explicit measures are better predictors for decided voters than implicit measures. The picture is, however, less clear if one looks at the incremental validity of the implicit measure. Interestingly, implicit attitudes did not predict voting intention for the minimum wage initiative beyond the explicit measure. They decreased Nagelkerke’s *R*^2^ by 0.5 percentage points after controlling for the explicit measure (model 2) and they did not change the percentage of correctly classified cases. However, in the case of the Gripen referendum, implicit attitudes increased Nagelkerke’s *R*^2^ by 9.2 percentage points after accounting for the explicit measure, while the %CCC increased by 3.9 percentage points.

**Table 2:** Results of logistic regression for prediction of voting intention.

Step	Variable	B	*SE*	Wald	*p*	Exp(B)	Nagel-kerke’s R^2^	%CCC
Minimum wage initiative (N = 154)								
1a	Constant	.263	.185	1.425	.154	1.301	.167	62.8%
	ST-IAT	.780	.200	3.902	<.001	2.181		
1b	Constant	.117	.215	.546	.585	1.124	.511	81.8%
	Explicit measure	−2.057	.359	−5.730	<.001	.128		
2	Constant	.197	.227	.868	.385	1.218	.506	81.8%
	ST-IAT	.306	.241	1.269	.204	1.358		
	Explicit measure	−1.781	.367	−4.849	<.001	.168		
Gripen referendum (N = 163)								
1a	Constant	−1.885	.295	−6.393	<.001	.152	.321	85.1%
	ST-IAT	1.442	.300	4.802	<.001	4.229		
1b	Constant	−1.727	.260	−6.638	<.001	.178	.398	85.3%
	Explicit measure	1.419	.245	5.798	<.001	4.131		
2	Constant	−1.990	.319	−6.241	<.001	.137	.490	89.2%
	ST-IAT	1.015	.324	3.130	.002	2.760		
	Explicit measure	1.168	.285	4.098	<.001	3.216		

Notes: B = regression weight; *SE* = standard error of the regression weight; Wald = Wald criterion; Exp(B) = Odds ratio, the relative amount by which the odds increase (Exp(B) > 1.0) or decrease (Exp(B) < 1.0) when the value of the predictor is increased by 1 unit; CCC = correctly classified cases; DV = voting intention (0 = No, 1 = Yes). All continuous variables were z-standardized prior to the analyses.

Table 3 depicts the results for the prediction of voting behavior. As for voting intention, the explicit measure better predicted vote choice in the case of the minimum wage initiative. They allowed for the correct classification of 81.4 percent of cases compared to only 69.4 percent of cases that were correctly predicted by implicit attitudes. However, for the Gripen referendum, the implicit measure did a slightly better job than the explicit measure in terms of correctly classified cases. It correctly predicted 82.4 percent of voters while the explicit measure did so for 81.4 percent of voters. Finally, for the latter referendum, adding the implicit to the explicit measure led to an increase in Nagelkerke’s *R*^2^ by 8.7 percentage points and an increase in the %CCC of 1.0 percentage points. In contrast, implicit attitudes did not predict voting on the minimum wage initiative beyond the explicit measure (−2.2 %CCC).

**Table 3:** Results of logistic regression for prediction of voting behavior.

Step	Variable	B	*SE*	Wald	*p*	Exp(B)	Nagel-kerke’s R^2^	% CCC
Minimum wage initiative (N = 86)								
1a	Constant	.356	.265	1.345	.179	1.400	.243	69.4%
	ST-IAT	1.051	.323	3.256	.001	2.860		
1b	Constant	.262	.298	.879	.379	1.300	.555	81.4%
	Explicit measure	−2.167	.468	−4.630	<.001	.115		
2	Constant	.073	.363	.200	.841	1.075	.615	79.2%
	ST-IAT	.405	.407	.994	.320	1.499		
	Explicit measure	−2.487	.697	−3.568	<.001	.083		
Gripen referendum (N = 86)								
1a	Constant	−1.891	.419	−4.517	<.001	.151	.207	82.4%
	ST-IAT	1.105	.416	2.658	.008	3.019		
1b	Constant	−1.847	.374	−4.942	<.001	.158	.327	81.4%
	Explicit measure	1.300	.332	3.913	<.001	3.669		
2	Constant	−2.199	.516	−4.265	<.001	.111	.414	82.4%
	ST-IAT	.842	.463	1.820	.069	2.322		
	Explicit measure	1.174	.399	2.939	.003	3.234		

Notes: B = regression weight; *SE* = standard error of the regression weight; Wald = Wald criterion; Exp(B) = Odds ratio, the relative amount by which the odds increase (Exp(B) > 1.0) or decrease (Exp(B) < 1.0) when the value of the predictor is increased by 1 unit; CCC = correctly classified cases; DV = voting behavior (0 = No, 1 = Yes). All continuous variables were z-standardized prior to the analyses.

In sum, the results of Study 1 support findings and claims made in previous research on the predictive value of implicit attitudes. First, they suggest that explicit indicators are better predictors for decided voters than implicit attitudes. In three out of four analyses, the explicit measure was—on its own—a better predictor of voting intention among decided respondents or voting behavior among those who had already voted. Second, and in line with the expectations made in previous research [10, 22], implicit-explicit consistency was considerably lower than in studies dealing with implicit attitudes toward political parties or camps. However, it seems that at least for the minimum wage initiative, implicit-explicit correspondence (Decided: *r* = −.37, Already voted: *r* = −.48) may still have been too high for the implicit measure to predict beyond the explicit measure.

One major limitation of Study 1 was the use of a WebInquisit ST-IAT which required participants to download a software plug-in. Unsurprisingly, downloading software raises concerns about malware and computer viruses among participants and thus acts as an effective impediment to data collection. In fact, the response rate in Study 1 was dramatically low (5.4%). For this reason, I used a computer-administered paper-format ST-IAT (CA-PF ST-IAT) in Study 2 that did not require respondents to download additional software.

## Study 2

Between September 22, 2014 and September 27, 2014, I ran a second study using a computer-administered paper-format ST-IAT to measure participants’ implicit attitudes toward the initiative “for a single health insurer.” The initiative was launched by the Social Democratic Party (SP) and aimed at replacing the competitive system of health insurance by a single public health insurer. Voting took place on September 28, 2014. The initiative was rejected by a 61.9 percent majority.

### Methods

#### Ethics statement

The same declaration applies as in the first study.

#### Participants

351 (42.7% males, 57.3% females) students, doctoral students, and employees of the University of Zurich volunteered for this study. Due to the different format of the ST-IAT, and thus the lack of appropriate information, no power analysis was conducted to determine sample size. However, since paper-format IATs tend to produce weaker IAT effects [23], I made sure that sample size was larger than in Study 1. Participants were told that the study was about the upcoming federal votes and asked to take the survey only if they were eligible to vote. Their mean age was 26.79 years (SD = 9.30).

#### Procedure

The Qualtrics (www.qualtrics.com) survey software was used to create the study. Participants answered the same questionnaires as in Study 1, along with two ST-IATs: a practice ST-IAT for implicit attitudes toward animals, and an ST-IAT for assessing their implicit attitudes toward the initiative for public health insurance.

#### Measures

Given the goal of overcoming the software plug-in impediment described above, I created an ST-IAT in line with Lemm et al.’s [23] suggestions. Acknowledging the fact that computer-format IATs are sometimes not feasible, Lemm and colleagues developed an IAT that can be administered with paper and pencil and that mimics the results and psychometric properties of computer-based counterparts. Furthermore, they tested various scoring algorithms and made convincing recommendations as to which algorithm should be used.

Both traditional computer IATs and paper-format IATs build on the assumption that the degree of congruence between a task and the implicit attitude of an individual determines the ease with which an individual can solve the task. In other words, if the pairing of a target with some evaluative category (e.g., positive) corresponds to the true implicit attitude of the individual, it will be easier for her to sort stimuli in this task than when target and evaluative category are reversed. However, unlike traditional computer IATs, paper-format IATs do not measure response latencies. Instead, they measure the number of correct categorizations within a given time period. For example, in Lemm et al.’s [23] studies, subjects were asked to categorize as many items as possible within a timeframe of 20 seconds. They were given a sheet with two columns of 20 items (i.e., trials) and asked to categorize items as either black or white names and pleasant or unpleasant words by marking the corresponding circles to the left and to the right of each item. After participants had completed the first condition, they received another 20 seconds for completing the second condition (in which category pairings were switched).

Building on the work of Lemm and colleagues [23], I created a computer-administered paper-format ST-IAT (CA-PF ST-IAT) for assessing participants’ implicit attitudes toward the initiative for public health insurance. As recommended by Lemm et. al [23], subjects first completed a training ST-IAT for assessing their implicit attitudes toward animals. Both training and critical ST-IAT consisted of six individual pages.

1. Participants were instructed to sort positive items by clicking the circle to the left of the item, while sorting negative items and items pertaining to the target category by clicking the right circle.
2. Once they felt ready to start, they could click on “Continue.” On the next page, a countdown of five seconds appeared along with the message “The task will start in 5 seconds! Make yourself ready!”
3. Subjects categorized as many items as possible from a list of 25 items within a fixed time period of 20 seconds. A countdown timer was visible at the top of the page. After time had run out, the survey automatically advanced to the next page.
4. Instructions for the second combined block (Positive or Target vs. Negative) were provided.
5. Before starting the task, subjects were again given five seconds to prepare.
6. Participants categorized as many items as possible from a list of 25 items, again within 20 seconds.

As in the previous study, block order was kept constant such that each participant first received the positive versus the negative or referendum condition (see S2 Appendix). In contrast to the previously administered ST-IAT versions, but in line with Lemm et al.’s [23] suggestions, only word stimuli were used (see S3 Appendix). Testing both paper-format IATs with verbal and picture stimuli and paper-format IATs with only verbal stimuli, Lemm and colleagues [23] found stronger correlations between the latter and traditional computer IATs. Moreover, test-retest reliability was better in the case of paper-format IATs using only verbal stimuli. For the evaluative categories, the same stimuli were used as in Study 1. The target category was represented by the names of two advocates (e.g., Jacqueline Fehr) of the initiative and the names of three parties (e.g., Green Party) supporting the initiative. ST-IAT scores were calculated using Lemm et al.’s [23] *product: square root of difference (PSQoD)* approach. Comparing seven different algorithms, they found that *PSQoD* is most consistent with results from computer-format IATs. If A denotes the number of correct responses in the first block and B the number of correct responses in the second block, then *PSQoD* is calculated as …

$$\begin{array}{c} \left(X/Y\right)\cdot \sqrt{X-Y}, \end{array}$$ (1)

where *X* is the greater of *A* or *B*, and *Y* is the smaller of *A* or *B*. Similar to Lemm et al. [23], only participants with at least six responses in both blocks were retained for data analyses. This resulted in the exclusion of 10 participants and thus a sample of 341 participants. As can be seen above, *PSQoD* takes both the difference between the number of correct responses (*X* − *Y*) and the ratio of correct responses (*X*/*Y*) into account. In addition, it controls for extreme scores by taking the square root of the difference between *X* and *Y*. Note that if an equal number of correct categorizations are made in both blocks, the resulting ST-IAT score will equal zero and thus indicate indifferent implicit attitudes. Finally, in order to retain the directionality of the ST-IAT effect, the resulting values were multiplied by −1 if *A* was greater than *B* (thus indicating a negative attitude toward the referendum of interest).

As in the first Study, participants’ self placement on the left-right scale was used as the explicit measure. The explicit measure was strongly correlated with voting for the public health insurance initiative (*r* = −.56, *p* < .001).

Voting intention and voting behavior were measured using the same questions as in Study 1. Undecided voters (N = 17) were again excluded from the analyses. This left 93 decided participants for the analysis of voting intention. Furthermore, there were no abstentions among those who had already voted on the referendum (N = 231).

### Results and Discussion

#### Preliminary analyses

Because paper (ST-)IATs do not rely on trial by trial measurement of response latencies, they do not allow for the calculation of split-half or alpha reliability. I can thus not report these statistics. Implicit-explicit consistency was moderate (*r* = −.38) and thus very similar to the correlations found in the first study. As in the previous study, implicit-explicit correspondence did not differ between participants who had already voted on the initiative (*r* = −.43, *p* < .001) and those who simply reported their voting intention (*r* = −.32, *p* < .001; *z* = −1.09, *p* of the difference between correlations = .28).

#### Voting intention and voting behavior

Tables 4 and 5 report the results from the logistic regression analyses for the prediction of voting intention and voting behavior. As in the first study, the implicit attitude measure predicted voting intention and vote choice, yet it was a worse predictor than participants’ self-reported (i.e., explicit) left-right placement. The explicit measure predicted 73.9 percent of decided voters correctly (Table 4) while the implicit measure could only predict 57.0 percent of cases. Similarly, implicit attitudes classified 66.5 percent of those respondents who had already voted correctly, while the explicit measure allowed for the correct classification of 73.9 percent of cases. Thus, as in the previous study, the explicit measure was a better predictor for decided voters.

**Table 4:** Results of logistic regression for prediction of voting intention.

Step	Variable	B	*SE*	Wald	*p*	Exp(B)	Nagel-kerke’s R^2^	% CCC
Public health insurance initiative (N = 93)								
1a	Constant	−.104	.216	−.480	.631	.901	.102	57.0%
	ST-IAT	.609	.242	2.519	.012	1.838		
1b	Constant	−.169	.257	−.658	.511	.845	.393	73.9%
	Explicit measure	−1.506	.343	−4.395	<.001	.222		
2	Constant	−.164	.260	−.630	.528	.849	.412	73.9%
	ST-IAT	.369	.275	1.340	.180	1.446		
	Explicit measure	−1.423	.346	−4.116	<.001	.241		

Notes: B = regression weight; *SE* = standard error of the regression weight; Wald = Wald criterion; Exp(B) = Odds ratio, the relative amount by which the odds increase (Exp(B) > 1.0) or decrease (Exp(B) < 1.0) when the value of the predictor is increased by 1 unit; CCC = correctly classified cases; DV = voting intention (0 = No, 1 = Yes). All continuous variables were z-standardized prior to the analyses.

**Table 5:** Results of logistic regression for prediction of voting behavior.

Step	Variable	B	*SE*	Wald	*p*	Exp(B)	Nagel-kerke’s R^2^	% CCC
Public health insurance initiative (N = 230)								
1a	Constant	.263	.141	1.866	.062	1.301	.149	66.5%
	ST-IAT	.756	.158	4.788	<.001	2.130		
1b	Constant	.239	.161	1.490	.136	1.270	.395	73.9%
	Explicit measure	−1.471	.202	−7.290	<.001	.230		
2	Constant	.242	.162	1.492	.136	1.274	.406	74.2%
	ST-IAT	.326	.186	1.755	.079	1.386		
	Explicit measure	−1.337	.209	−6.394	<.001	.263		

Notes: B = regression weight; *SE* = standard error of the regression weight; Wald = Wald criterion; Exp(B) = Odds ratio, the relative amount by which the odds increase (Exp(B) > 1.0) or decrease (Exp(B) < 1.0) when the value of the predictor is increased by 1 unit; CCC = correctly classified cases; DV = voting behavior (0 = No, 1 = Yes). All continuous variables were z-standardized prior to the analyses.

Finally, implicit attitudes did not improve model fit in the case of voting intention. Adding the implicit measure to the explicit measure (model 2) only resulted in a slight increase in Nagelkerke’s R2 (+1.9 percentage points) and no change in the %CCC. Finally, the implicit measure did not substantially improve the quality of the prediction of voting behavior (+0.3 %CCC) once the explicit measure was controlled for.

To summarize, both studies suggest that implicit attitudes predict voting intention and voting behavior in Swiss referendums. However, at least for decided voters, they are worse predictors than explicit measures such as self-reported left-right placement. More importantly, although the correlations between the implicit and explicit measure were generally lower than in studies on elections (e.g., [10]), implicit attitudes did only provide little incremental validity (between 0.3 and 1.0 percentage points) and in one case (i.e., the minimum wage initiative) they even decreased the %CCC. However, studies 1 and 2 were limited in scope to decided voters. Hence, they could not address the claim made in previous research (e.g., [9]) that implicit attitudes are better predictors for undecided voters as compared to decided voters. To evaluate this claim, I conducted an additional study. Because of the more pragmatic (i.e., more user-friendly) character of the computer-administered paper-format ST-IAT, I used this particular test version in a third study combining both a pre- and post-vote survey.

## Study 3

Study 3 took place three weeks ahead of the national vote (November 30, 2014) on the so-called “Ecopop initiative.” Launched by the organization Ecology and Population (ECOPOP), the referendum proposed limiting the annual net migration to 0.2 percent of Switzerland’s resident population. Unlike most other anti-immigration referendums, the Ecopop initiative did not refer to immigration as a threat to Swiss society or its economy but rather as a major cause of environmental degradation. It was thus believed to attract votes from both right-wing voters and, to a lesser degree, voters identifying with the Green and Alternative Left Parties. Due to its likely negative impact on the Swiss economy and its xenophobic character, the referendum faced opposition from all major political parties and the Swiss government. It was eventually rejected by a clear majority of 74.1 percent.

### Methods

#### Ethics statement

The study was conducted online. All subjects were older than 18 years of age. Data collection was performed by respondi AG which conforms to the ESOMAR codes and guidelines for online access panels. On the first page, subjects received brief information on the purpose of the study, the measures involved, and the leading research institute (Department of Political Science, University of Zurich). At the end of the study, participants received contact information. Debriefing was provided on request. All data was analyzed anonymously and no identifying data (e.g., names) were collected. At the author’s faculty (Faculty of Arts, University of Zurich) the IRB asks researchers to self-assess the ethical soundness of their research using a checklist and only seek approval from the IRB if one of the questions is answered affirmatively. This was not the case for the present study.

#### Participants

Sampling was done using the online access panel provided by respondi AG. Only eligible voters (N = 1824) from the German- and French-speaking parts of Switzerland were recruited. Retaining participants with at least six correct responses in the ST-IAT blocks resulted in the exclusion of 183 participants. Of the remaining 1641 participants, 1006 participants (37.48% males, 62.52% females) reported that they had not yet voted and 862 participants (38.98% males, 61.02% females) answered affirmatively when asked whether they intended to vote. Of those, 704 returned for the second survey, which took place in the week after the vote (return rate of 81.67%, 41.05% males, 58.95% females), and 552 eventually reported their vote choice (44.38% males, 55.62% females). Mean age among participants in the final sample was 43.35 years (SD = 13.87). Participants received a small compensation of EUR 1.10 for participation in the first survey and EUR 0.50 for participation in the second survey. The final sample consisted of 457 participants who initially reported to be decided and 82 participants who reported to be undecided. Due to the low return rate among undecided voters (49.10%), the final sample of undecided voters was slightly smaller than the one determined a priori using G*Power [24], a stand-alone power analysis program for statistical tests. The analysis indicated that for the desired power (.80), alpha level (.05), anticipated (i.e., based on Study 2) effect size (OR = 2.0), and two moderately correlated predictors (R^2^ other X = 0.15), a minimum sample size of 97 was required.

#### Procedure

In the pre-vote survey, participants completed several questionnaires on socio-demographic variables, political variables (e.g., partisanship, left-right placement), and their concern about the impact of immigration on the economy, culture, and environment. At the end of the survey, they completed the same practice ST-IAT used in the previous study, followed by another ST-IAT for assessing their implicit attitude toward “Ecopop.” After voting day, subjects received an invitation to take the second survey, in which they were asked to report their vote choice. Both surveys were programmed in Qualtrics.

#### Measures

Implicit attitudes were measured using the same ST-IAT as in the previous study. For the target category (i.e., Ecopop initiative), I used the names of three politicians, one right-wing party, and one political association supporting the referendum. In order to account for differences between language regions, I used slightly different stimuli in the French version of the ST-IAT than in the German ST-IAT (see S4 Appendix).

Due to the anti-immigration character of the Ecopop initiative, explicit attitudes were assessed with three items that asked participants to indicate their concern about the impact of immigration on the (i) economy, (ii) Swiss culture, and (iii) environment using 6-point Likert items (1 = *very unconcerned* to 6 = *very concerned*) (*α* = 0.86). Items were averaged to form the concern scale, with high numbers reflecting higher concern about the impact of immigration. Using concern about the impact of immigration instead of self-reported evaluations of Ecopop was similar to the approach of Galdi et al. [9] who used multiple items to measure participants’ conscious beliefs about the consequences of the enlargement of a U.S. military base. Voting on Ecopop was strongly associated (*r* = .49, *p* < .001) with explicit attitudes.

Voting intention and voting behavior were measured in the same way as in Studies 1 and 2. In the pre-vote survey, 692 participants reported their vote intention and 167 were undecided. In the second survey (i.e., after the vote), 13 of 552 participants reported that they had abstained from the vote. This left a total of 539 participants for the analyses of voting behavior.

### Results and Discussion

#### Preliminary Analyses

I first tested for consistency between the implicit and explicit measure for both decided and undecided voters. Overall, correspondence between the implicit and explicit measure was lower (*r* = .23) than in the previous studies. In addition, the two types of measures were only correlated among decided voters (*r* = .27, *p* < .001) but not among undecided voters (*r* = .01, ns; *z* = 3.06, *p* of the difference between correlations <.01). These results are consistent with previous research (e.g., [10]) arguing that consistency between implicit and explicit attitudes is more pronounced among decided voters most likely as a result of their greater cognitive elaboration of attitudes. In sum, implicit-explicit correspondence was relatively low and closer to the correlations found in Galdi et al. [9] than in pre-election studies (e.g., [10]).

#### Voting intention

On their own, both the implicit (model 1a) and explicit (model 1b) measure predicted voting intention (see Table 6). However, as in the previous studies, the explicit measure better predicted voting intention. Moreover, looking at the incremental validity of implicit attitudes, I found that the latter increased Nagelkerke’s R^2^ by 1.8 percentage points after controlling for explicit attitudes (model 2) but decreased the percentage of correctly classified cases by 0.4 percentage points. On the other side, the explicit measure increased Nagelkerke’s R^2^ by 29.0 percentage points after accounting for implicit attitudes, while correctly classified cases increased by 8.2 percentage points. In sum, the explicit measure performed better in terms of both predictive validity in general and incremental validity.

**Table 6:** Results of logistic regression for prediction of voting intention.

Step	Variable	B	*SE*	Wald	*p*	Exp(B)	Nagel-kerke’s R^2^	% CCC
Ecopop initiative (N = 691)								
1a	Constant	−.607	.083	−7.343	<.001	.545	.090	66.0%
	ST-IAT	.572	.088	6.467	<.001	1.771		
1b	Constant	−.835	.101	11.716	<.001	.434	.362	74.6%
	Explicit measure	1.460	.125	−8.296	<.001	4.307		
2	Constant	−.844	.102	−8.187	<.001	.430	.380	74.2%
	ST-IAT	.357	.102	3.506	<.001	1.429		
	Explicit measure	1.378	.126	10.943	<.001	3.969		

Notes: B = regression weight; *SE* = standard error of the regression weight; Wald = Wald criterion; Exp(B) = Odds ratio, the relative amount by which the odds increase (Exp(B) > 1.0) or decrease (Exp(B) < 1.0) when the value of the predictor is increased by 1 unit; CCC = correctly classified cases; DV = voting intention (0 = No, 1 = Yes). All continuous variables were z-standardized prior to the analyses.

#### Voting behavior

Table 7 below presents results from binary logistic regression models for the prediction of voting behavior among decided and undecided voters. Implicit (model 1a) and explicit (model 1b) attitudes both predicted voting behavior of decided and undecided voters. However, a comparison of the implicit-only models (models 1a) for decided and undecided voters reveals that, on their own, implicit attitudes are better predictors of voting behavior among undecided voters, with 78.1 percent of them being correctly classified (as compared to 73.0 percent among decided voters). In line with these descriptive results, implicit attitudes improved model fit more for undecided voters than for decided voters once explicit attitudes were controlled for. In fact, Nagelkerke’s R^2^ increased by only 1.1 percentage points for decided voters, while it increased by 5.5 percentage points for undecided voters upon adding the implicit measure to the explicit measure (model 2). However, in regards to the percentage of correctly classified cases, implicit attitudes improved model fit to an equal extent for decided (+1.1 %CCC) and undecided (+1.2 %CCC) voters. On the other side, explicit attitudes increased Nagelkerke’s R^2^ by 32.0 percentage points and 13.0 percentage points for decided and undecided voters after accounting for implicit attitudes, while correctly classified cases increased by 6.8 and 0.9 percentage points for decided and undecided voters. Taken together, from a descriptive point of view, explicit attitudes better predicted vote choice of decided voters, while implicit attitudes fared slightly better than explicit attitudes in their prediction of vote choice among undecided voters.

**Table 7:** Results of logistic regression for prediction of voting behavior, separately for decided and undecided voters.

Step	Variable	B	*SE*	Wald	*p*	Exp(B)	Nagel-kerke’s R^2^	% CCC
Decided voters who voted (N = 457)								
1a	Constant	−1.003	.110	−9.127	<.001	.367	.081	73.0%
	ST-IAT	.593	.121	4.899	<.001	1.809		
1b	Constant	−1.380	.146	−9.421	<.001	.252	.390	78.7%
	Explicit measure	1.574	.166	9.511	<.001	4.827		
2	Constant	−1.390	.148	−9.410	<.001	.249	.401	79.8%
	ST-IAT	.335	.139	2.408	.016	1.399		
	Explicit measure	1.491	.168	8.887	<.001	4.440		
Undecided voters who voted (N = 82)								
1a	Constant	−1.378	.292	−4.727	<.001	.252	.082	78.1%
	ST-IAT	.644	.322	1.997	.046	1.904		
1b	Constant	−1.514	.323	−4.694	<.001	.220	.157	77.8%
	Explicit measure	.977	.360	2.712	.007	2.657		
2	Constant	−1.635	.353	−4.628	<.001	.195	.212	79.0%
	ST-IAT	.619	.358	1.731	.083	1.857		
	Explicit measure	.948	.367	2.580	.010	2.580		

Notes: B = regression weight; *SE* = standard error of the regression weight; Wald = Wald criterion; Exp(B) = Odds ratio, the relative amount by which the odds increase (Exp(B) > 1.0) or decrease (Exp(B) < 1.0) when the value of the predictor is increased by 1 unit; CCC = correctly classified cases; DV = voting behavior (0 = No, 1 = Yes). All continuous variables were z-standardized separately for decided and undecided voters prior to the analyses.

To complement these descriptive analyses, I conducted moderation analyses to further examine the potential interaction between the implicit and explicit measure and decidedness. Table 8 shows that, when modeled separately, neither the explicit (model 1) nor the implicit measure (model 2) predicted voting behavior significantly better for decided individuals. Although both coefficients point in the expected direction, they do not reach conventional levels of significance. The same can be said when both implicit and explicit attitudes are considered along with decidedness and their respective interactions (model 3). Thus, implicit attitudes predicted voting behavior descriptively, but not significantly better for undecided voters while explicit attitudes predicted voting behavior descriptively, but not significantly better for decided respondents.

**Table 8:** Results of logistic regression analyses predicting voting behavior from explicit (EA) and implicit attitudes (IA) and decidedness.

Model	Variable	B	*SE*	Wald	*p*	Exp(B)	Nagel-kerke’s R^2^	% CCC
1	Explicit attitudes (N = 532)							
	Constant	−1.581	.335	−4.719	<.001	.206	.361	78.6%
	EA	1.101	.406	2.712	.007	3.007		
	Decidedness	.227	.365	.622	.534	1.255		
	EA*Decidedness	.512	.440	1.164	.245	1.669		
2	Implicit attitudes (N = 538)							
	Constant	−1.452	.306	−4.744	<.001	.234	.084	73.8%
	IA	.619	.310	1.997	.046	1.858		
	Decidedness	.437	.325	1.342	<.001	.180	1.548	
	IA*Decidedness	−.020	.333	−.058	.953	.981		
3	Explicit and implicit attitudes (N = 531)							
	Constant	−1.771	.382	−4.638	<.001	.170	.377	79.7%
	EA	1.068	.414	2.580	.010	2.910		
	IA	.595	.344	1.731	.083	1.814		
	Decidedness	.399	.409	.974	.330	1.490		
	EA*Decidedness	.459	.448	1.025	.305	1.583		
	IA*Decidedness	−.256	.372	−.689	.491	.774		

Notes: B = regression weight; *SE* = standard error of the regression weight; Wald = Wald criterion; Exp(B) = Odds ratio, the relative amount by which the odds increase (Exp(B) > 1.0) or decrease (Exp(B) < 1.0) when the value of the predictor is increased by 1 unit; CCC = correctly classified cases; DV = voting behavior (0 = No, 1 = Yes). All continuous variables were z-standardized prior to the analyses.

## General Discussion

The use of implicit attitude measures has been proposed to tackle the challenge of predicting the vote of undecided voters. Most of the optimism has stemmed from a seminal study [9] in which implicit, but not explicit, attitudes were found to be significant predictors of undecided respondents’ opinions on a political issue. Interestingly, the pattern of results was reversed for decided respondents: Explicit, but not implicit, attitudes predicted future opinions. Against the backdrop of these results, a full double dissociation pattern [9, 25] has been proposed comprising four hypotheses:

1. Implicit attitudes predict voting behavior better than explicit attitudes for undecided voters.
2. Explicit attitudes predict voting behavior better than implicit attitudes for decided voters.
3. Implicit attitudes predict voting behavior better for undecided than decided voters.
4. Explicit attitudes predict voting behavior better for decided than undecided voters.

However, recent studies have shed doubt on the incremental validity of implicit attitudes in general and their utility for undecided voters in particular. Reviewing the more recent evidence on the predictive value of implicit attitudes, Friese et al. [22] conclude that [i]n most cases […], the increase in %CCC remained well below 1 percentage point, sometimes there was no change at all, and sometimes even descriptively a decrease in %CCC occurred. (p. 19) What is more, the authors also mention that there is […] no evidence for the ideas that implicit measures predict the voting behavior of undecided voters (a) better than explicit measures do, or (b) better than they predict the behavior of decided voters. (p. 19) In an attempt to explain why replications of the double dissociation pattern have generally failed, Friese et al. [22] point to the contextual differences between the seminal study by Galdi et al. [9] and subsequent research. In fact, while the former dealt with implicit attitudes toward a local political issue where no actual voting took place, subsequent studies have focused on real political elections and voting behavior. In such a context, implicit and explicit measures tend to overlap as a result of well elaborated attitudes toward parties or candidates and this will, eventually, leave little room for implicit measures to predict beyond explicit measures.

It follows from the above reasoning that implicit measures may be better predictors in the context of voting on specific political issues where attitudes tend to be less elaborated. For this reason, I have tested the predictive validity of implicit attitudes in the context of issue-related votes in Switzerland. In the remainder of this section, I shall briefly discuss my results with respect to the four hypotheses presented above.

**1. Implicit attitudes predict voting behavior better than explicit attitudes for undecided voters.** Only the design of the third study allowed for a comparison between decided and undecided voters. Unlike stated in the hypothesis, implicit and explicit attitudes were equally good predictors of vote choice among undecided voters. On their own, implicit attitudes correctly predicted the vote of 78.1 percent of undecided respondents while explicit attitudes did so for 77.8 percent.

**2. Explicit attitudes predict voting behavior better than implicit attitudes for decided voters.** All three studies support this claim. In Studies 1 and 2, self-reported left-right placement was a better predictor of participants’ voting intention (minimum wage initiative: IA: 62.8 %CCC, EA: 81.8 %CCC; Gripen referendum: IA: 85.1 %CCC, EA: 85.3 %CCC; public health insurance initiative: IA: 57.0 %CCC, EA: 73.9 %CCC) and in Study 3 participants’ concern about immigration better predicted voting behavior among decided voters (IA: 73.0 %CCC, EA: 78.7 %CCC).

**3. Implicit attitudes predict voting behavior better for undecided than decided voters.** In Study 3, I found some, albeit descriptive, evidence for this hypothesis. The implicit measure was a better predictor for the voting behavior of undecided (78.1 %CCC) than decided (73.0 %CCC) voters. However, implicit attitudes improved the quality of the overall prediction only slightly for both decided (+1.1 %CCC) and undecided voters (+1.2 %CCC) and there was no significant interaction between implicit attitudes and decidedness.

**4. Explicit attitudes predict voting behavior better for decided than undecided voters.** Consistent with this hypothesis, explicit attitudes better predicted voting behavior among decided voters (78.7 %CCC) than among undecided voters (77.8 %CCC). In addition, they improved model fit to a greater extent for decided voters (+6.8 %CCC) than undecided voters (+0.9 %CCC). Nevertheless, these descriptive analyses were not backed up by a subsequent moderation analysis.

Taken together, my results stand in-between those of Friese and colleagues [10] and those of Galdi et al. [9]. As in the former study, explicit measures were better predictors of voting behavior among decided voters. However, unlike results of Friese et al. [10] but similar to Galdi et al. [9], implicit attitudes did, at least from a descriptive perspective, a better job in predicting choices of undecided voters as compared to decided voters. Yet, unlike in Galdi and colleagues study, they did not outperform explicit attitudes. Thus, neither the results of Galdi and colleagues nor the results of Friese and colleagues could be replicated. Given that the correspondence between the implicit and explicit measure was fairly low in all three studies (*r* = .23 – .40), it is surprising that the double dissociation pattern described above could not be replicated. Researchers should, however, keep in mind that the cognitive elaboration of attitudes may not necessarily represent the only moderator of the predictive validity of implicit measures. For example, research on attitudinal ambivalence suggests that ambivalent individuals rely less on their implicit attitudes when making decisions. Hence, implicit attitudes should be less relevant for ambivalent, and thus undecided, voters. This example shows that a multitude of theoretical models and mechanisms exists explaining why and under what conditions implicit attitudes should predict voting behavior of undecided individuals. Researchers will need to rethink some of these theoretical accounts while, at the same time, test others before implicit attitudes will allow for considerable improvements of political predictions.

## Supporting Information

S1 Appendix Stimuli list for ST-IATs on minimum wage initiative and Gripen referendum (Study 1). (PDF)

S2 Appendix First condition of computer-administered paper-format ST-IAT (Study 2). (PDF)

S3 Appendix Stimuli list for ST-IAT on public health insurance initiative (Study 2). (PDF)

S4 Appendix Stimuli list for ST-IAT on Ecopop initiative (Study 3). (PDF)
