# Supplementary material for: Single-Target Implicit Association Tests (ST-IAT) Predict Voting Behavior of Decided and Undecided Voters in Swiss Referendums
Source: PLoS One. 2016 Oct 12;11(10):e0163872. doi: 10.1371/journal.pone.0163872 (PMC5061388; doi:10.1371/journal.pone.0163872)
Supplement: S1 Appendix — (PDF) [file pone.0163872.s001.pdf]

Stimuli List for ST-IATs on Minimum Wage Initiative & Gripen Referendum (Study 1)

Category	Stimuli
positive	Love (Liebe), Joy (Freude), Paradise (Paradies), Gift (Geschenk), Holiday (Ferien)
negative	Poison (Gift), Stink (Gestank), Disease (Krankheit), Disaster (Katastrophe), Death (Tod)
Minimum Wage Initiative	Cédric Wermuth (words), Christian Levrat (words), Campaign Poster (picture), SP party emblem (picture), UNIA emblem (picture)
Gripen Referendum	Schweizer Armee (words), Ueli Maurer (words), Campaign Poster (picture), Gripen fighter jet (picture), André Blattmann (photo)

Note: Original German positive and negative stimuli are in brackets.Target stimuli were words and pictures.
