# Supplementary material for: Single-Target Implicit Association Tests (ST-IAT) Predict Voting Behavior of Decided and Undecided Voters in Swiss Referendums
Source: PLoS One. 2016 Oct 12;11(10):e0163872. doi: 10.1371/journal.pone.0163872 (PMC5061388; doi:10.1371/journal.pone.0163872)
Supplement: S2 Appendix — (PDF) [file pone.0163872.s002.pdf]

First condition of computer-administered paper-format ST-IAT (Study 2)

Positive	Negative
	or
	Initiative for Public Health Insurance

○	Love	○
○	Joy	○
○	Jacqueline Fehr (SP)	○
○	SP	○
○	Paradise	○
○	Yvonne Gilli (Green)	○
○	Poison	○
○	Gift	○
○	Holidays	○
○	Green Party	○
○	EVP	○
○	Love	○
○	Stink	○
○	Joy	○
○	Jacqueline Fehr (SP)	○
○	SP	○
○	Disease	○
○	Disaster	○
○	Holidays	○
○	Gift	○
○	EVP	○
○	Paradise	○
○	Death	○
○	Green Party	○
○	Yvonne Gilli (Green)	○
