# Supplementary material for: Single-Target Implicit Association Tests (ST-IAT) Predict Voting Behavior of Decided and Undecided Voters in Swiss Referendums
Source: PLoS One. 2016 Oct 12;11(10):e0163872. doi: 10.1371/journal.pone.0163872 (PMC5061388; doi:10.1371/journal.pone.0163872)
Supplement: S3 Appendix — (PDF) [file pone.0163872.s003.pdf]

Stimuli List for ST-IAT on Public Health Insurance Initiative (Study 2)

Category	Stimuli
positive	Love (Liebe), Joy (Freude), Paradise (Paradies), Gift (Geschenk), Holiday (Ferien)
negative	Poison (Gift), Stink (Gestank), Disease (Krankheit), Disaster (Katastrophe), Death (Tod)
Public Health Insurance Initiative	Jacqueline Fehr (SP), SP, Yvonne Gilli (Grüne), Grüne Partei, EVP

Note: Original German positive and negative stimuli are in brackets. Target stimuli were only words.
