# Supplementary material for: Single-Target Implicit Association Tests (ST-IAT) Predict Voting Behavior of Decided and Undecided Voters in Swiss Referendums
Source: PLoS One. 2016 Oct 12;11(10):e0163872. doi: 10.1371/journal.pone.0163872 (PMC5061388; doi:10.1371/journal.pone.0163872)
Supplement: S4 Appendix — (PDF) [file pone.0163872.s004.pdf]

Stimuli List for ST-IAT on Ecopop Initiative (Study 3)

Category	Stimuli
positive	Love (Liebe/Amour), Joy (Freude/Joie), Paradise (Paradies/Paradis), Gift (Geschenk/Cadeau), Holiday (Ferien/Vacances)
negative	Poison (Gift/Poison), Stink (Gestank/Puanteur), Disease (Krankheit/Maladie), Disaster (Katastrophe/Catastrophe), Death (Tod/Mort)
Ecopop Initiative (German)	Thomas Minder, Schweizer Demokraten (SD), Hans Geiger (SVP), Aktion für eine Unabhängige und Neutrale Schweiz (AUNS), Pirmin Schwander (SVP)
Ecopop Initiative (French)	Thomas Minder, Démocrates Suisses (DS), Philippe Roch, Action pour une Suisse Indépandante et Neutre (ASIN), Pirmin Schwander (SVP)

Note: Original German and French positive and negative stimuli are in brackets. Target stimuli were only words.
